# Supplementary figures and images for: Neuropeptide S receptor gene Asn107 polymorphism in obese male individuals in Pakistan
Source: PLoS One. 2020 Dec 17;15(12):e0243205. doi: 10.1371/journal.pone.0243205 (PMC7745988; doi:10.1371/journal.pone.0243205)

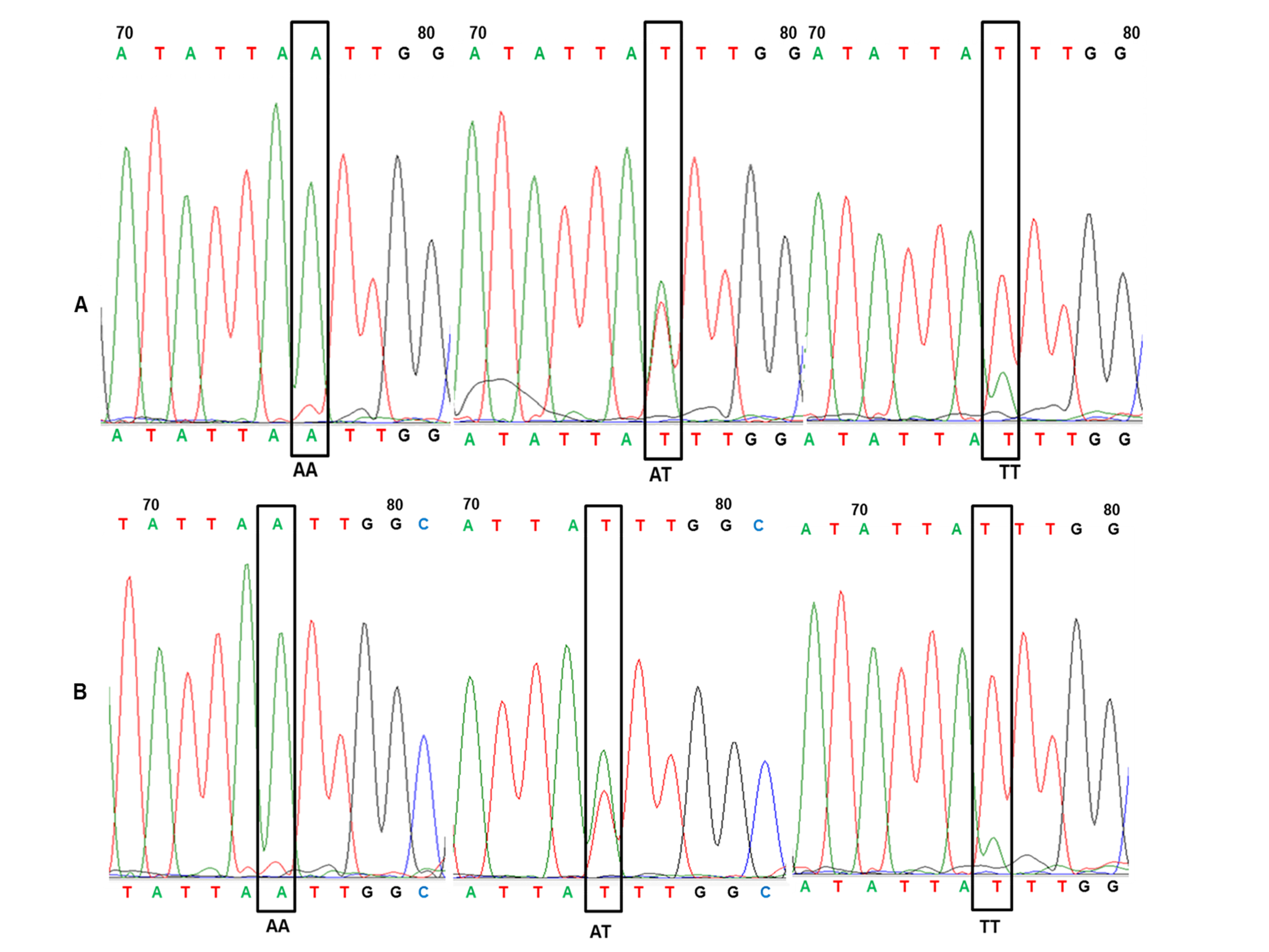

Supplement: S1 Fig — (TIF) [file pone.0243205.s001.tif]

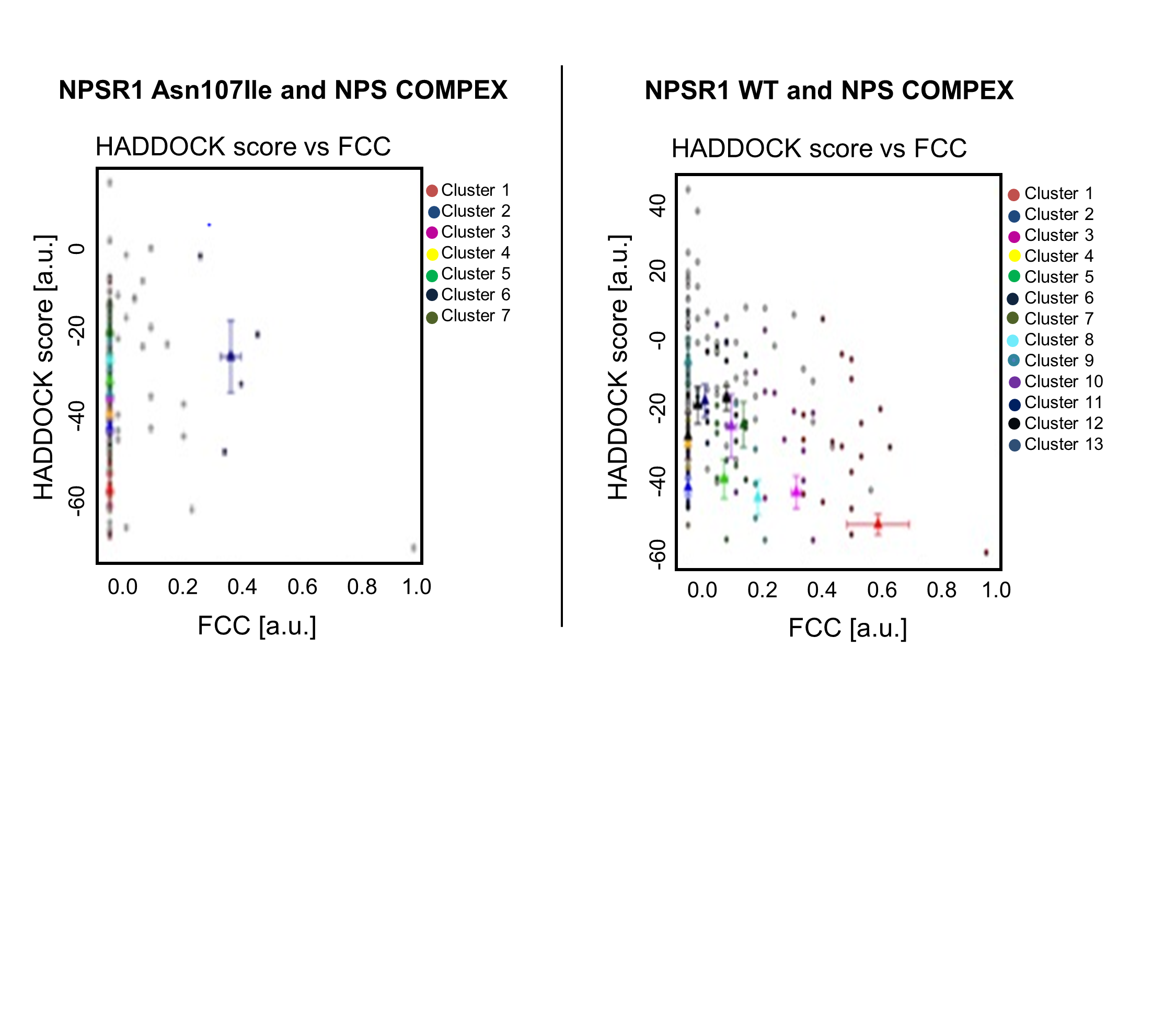

Supplement: S2 Fig — The higher the score the closer the interaction between NPS and NPSR1. In cluster 1 (red—circled) which includes residues (100–330). (TIF) [file pone.0243205.s002.tif]
