# Supplementary material for: Neuropeptide S receptor gene Asn107 polymorphism in obese male individuals in Pakistan
Source: PLoS One. 2020 Dec 17;15(12):e0243205. doi: 10.1371/journal.pone.0243205 (PMC7745988; doi:10.1371/journal.pone.0243205)
Supplement: S1 Table — (DOCX) [file pone.0243205.s003.docx]

**S1 Table.** **Moderation Effect Analysis,** when genotypes act as a moderator while obesity is independent variable and NPS is response variable.

| **Genotype Effect t-statisctics CI Lower-Upper level) P-value** |
| --- |
| Moderation AA -8.089 -6.583 95% ( -10.524 -5.654) P<0.000 |
| Moderation AT/TT -5.216 -7.085 95% (-6.675 -3.757 P<0.000 |
