# Supplementary material for: Neuropeptide S receptor gene Asn107 polymorphism in obese male individuals in Pakistan
Source: PLoS One. 2020 Dec 17;15(12):e0243205. doi: 10.1371/journal.pone.0243205 (PMC7745988; doi:10.1371/journal.pone.0243205)
Supplement: Attachment — Submitted filename: review.docx [file pone.0243205.s005.docx]

I read carefully version of the article titled “Neuropeptide S receptor gene Asn107 polymorphism in obese male individuals in Pakistan”

I have several major comments listed below ---

1. Neuropeptide S receptor (NPSR1) gene might have many SNPs, the author should describe the possible reason why they choose rs324981 to study.
2. The figures are too obscure to be seen. The authors should provide clearer graph.
3. The language and paper structure is too obscure to understand. Such as “**clinical picture** “. What is the meaning of clinical picture?
4. The sample size is too small for SNP polymorphism study.
