# Supplementary material for: Neuropeptide S receptor gene Asn107 polymorphism in obese male individuals in Pakistan
Source: PLoS One. 2020 Dec 17;15(12):e0243205. doi: 10.1371/journal.pone.0243205 (PMC7745988; doi:10.1371/journal.pone.0243205)
Supplement: Attachment — Submitted filename: Response to Reviewers.docx [file pone.0243205.s006.docx]

Response Letter addressing “Revision”

**Dear Editor PLOS ONE,**

**With Reference ;**

PONE-D-19-35919

Neuropeptide S receptor gene Asn107 polymorphism in obese male individuals in Pakistan

The Manuscript Revision comments & Response here for your consideration.The authors appreciate the time the reviewers have invested in reviewing our manuscript carefully and for their valuable input which has significantly enhanced the impact of this manuscript. Following are the responses to reviewer comments and subsequent changes in the revised manuscript.

The specific comments and their responses are mentioned below.

**Reviewer 1**

**Comment # 1**. **Neuropeptide S receptor (NPSR1) gene might have many SNPs, the author should describe the possible reason why they choose rs324981 to study.**

**Response.** NPS-NPSR1 interaction have been discussed in discussion section of manscript, this system on downstream accelerates MAPK activity and fatty acid oxidation to protect organisms against diet-induced obesity (Reinscheid et al., 2005; Anedda F., et al 2011). In cell models, the change of Asn(107) to Ile(107) results in 10-fold increase in NPS-mediated intracellular signaling (Bernier et al., 2006; Anedda et al., 2011). A nonsynonymous SNP at rs324981 is more functional gene variation among the well-known NPSR1 SNPs rs324987, rs324957, rs323920, rs324396 and rs323922 (Yan Feng, et al., 2006). GWAS have found an association of rs324981 with major obesity linked comorbidities like IBD, depression, anxiety, panic disorder, sleep and rest in diverse populations but still true causative variations remain to be identified (Gottlieb DJ., et al 2007; Reinscheid, 2008; Domschke et al., 2011; Lennertz L. et al., 2013).

**Comment # 2. The figures are too obscure to be seen. The authors should provide clearer graph.**

**Response:** We have revisited the all figures addressed the issues according to PLOS ONE figures guidelines https://journals.plos.org/plosone/s/figures.

**Comment # 3. The language and paper structure is too obscure to understand. Such as “clinical picture “. What is the meaning of clinical picture?**

**Response;** We have revisited the complete manuscript considering these critical comments and addressed them accordingly. There are number of sentences which have been rephrased for better expression. Moreover, language and grammar was thoroughly checked from abstract to conclusions sections and all the necessary technical expression have been amended accordingly.

a)	Language and grammar revised by

**Habib Bokhari, PhD**

Commonwealth Scholar & Fellow (LSHTM, UK)

Fulbright Fellow (Perelman School of Medicine, UPENN, USA)

Professor, Department of Biosciences COMSATS University, Islamabad, Pakistan

Office: +92-51-250-1223; FAX: +92-51-444-2805 Mobile: +92-300-512-7684

E-mail: habib@comsats.edu.pk

b)	A copy of manuscript showing track changes has been uploaded.

c)	A clean copy of the edited manuscript has been uploaded as the new manuscript file.

**Comment # 4. The sample size is too small for SNP polymorphism study.**

**Response;** This study has potential limitations because of limited resources availability, time constraint and stringent inclusion/exclusion criteria for both case & controls. However, the data does have statistical significance as described in the next comment. We have added discussion about this topic to the discussion.

**Reviewer # 2:**

**Comment # 1**. **For a genetic study, the sample size is too small.**

**Response;** This study has potential limitations because of limited resources availability, time constraint and stringent inclusion/exclusion criteria for both case & controls. However, the data does have statistical significance as described in the next comment. We have added discussion about this topic to the discussion.

**Comment # 2**. **According to the data given in the manuscript, the result of the chi-square test for genotypes is incorrect. The chi-square value should be 4.251, which does not reach a significant level (p=0.119), and the z-test was not significant either. It is not clear how chi-square=4.131 (p=0.04) is calculated. Therefore, in my opinion, there are no significant results in this study.**

**Response;** Actually we applied the Dominant model considering “T” as a dominant allele (Feifei Zhao et al., 2016). We have reordered data table accordingly.

**Obese	Control**	Row Total

**T**	29(60.42%)	19(39.58%)	48(100%)

A	28(41.18%)	40(58.82%)	68(100%)

Column Total	57	58	116

The chi-square statistic is 4.1677. The *p*-value is .041201. Significant at *p* < .05.

**Additional Editor Comments**

**The sample size is too small, so the authors should provide the statistical power. In addition, it should be discussed in the limitation.**

**Response;** This study has potential limitations because of limited resources availability, time constraint and stringent inclusion/exclusion criteria for both case & controls. However, the data does have statistical significance as described in the next comment. We have added discussion about this topic to the discussion.

**Comment # 1**. **Please ensure that your manuscript meets PLOS ONE's style requirements, including those for file naming.**

**Response;** Thanks for your suggestion, We have revised the all file names and style to conform closely to PLOS ONE's requirements.

**Comment # 2**. **Please upload a copy of Figure 8, to which you refer in your text on page 16. If the figure is no longer to be included as part of the submission please remove all reference to it within the text.**

**Response.** Yes, figure reference has been removed from the text and figure 8 is no longer part of submission.

**Comment # 3. Please upload a new copy of Figure S8 as the detail is not clear.**

**Response.** Thanks for your input, a new copy of Fig S8 labeled as Fig S7 with clear details has been uploaded.
