# Supplementary material for: Neuropeptide S receptor gene Asn107 polymorphism in obese male individuals in Pakistan
Source: PLoS One. 2020 Dec 17;15(12):e0243205. doi: 10.1371/journal.pone.0243205 (PMC7745988; doi:10.1371/journal.pone.0243205)
Supplement: Attachment — Submitted filename: Response to Reviewers.docx [file pone.0243205.s007.docx]

Response Letter addressing “Revision”

**Dear Editor PLOS ONE,**

**With Reference ;**

PONE-D-19-35919

Neuropeptide S receptor gene Asn107 polymorphism in obese male individuals in Pakistan

The Manuscript Revision comments & Response here for your consideration. The authors appreciate the time the reviewers have invested in reviewing our manuscript carefully and for their valuable input which has significantly enhanced the impact of this manuscript. Following are the responses to reviewer comments and subsequent changes in the revised manuscript.

The specific comments and their responses are mentioned below.

**Reviewer 2**

**Comment # 1**. **Comment1: The genotype of Asn107lle in this manuscript did not conform to the Hardy Weinberg Equation, this should be reported in the article. And the deviation of HWE may due to the small sample size, but for me, it much more like there have genotype errors, especially by using a PCR-RFLP method. The authors said they have confirmed the results by Sanger sequencing, how much samples have been confirmed, especially for Heterozygotes and Rare Homozygotes?**

**Response.** Yes, genotype of all 116 samples reported in this manuscript was confirmed by Sanger sequencing rather than relying on PCR-RFLP method, and sequencing results are submitted here as a “supplementary Sanger sequencing file”. Furthermore this genotype data follows the Hardy Weinberg Equation, calculations are shown below.

| Genotype | | | Allelic Frequency | |
| --- | --- | --- | --- | --- |
| AA | TT | AT | A | T |
| 68 | 24 | 24 | 0.69 | 0.31 |

*f(T)= (AT)+2 X (TT)/2 X (TT) + 2 X (24) + 2 X (AA)*

*=24+ 2(24)/2(24)+2(24)+2(68)*

*= 72/232*

*= 0.31*

*f(A)= (AT)+2 X (AA)/2 X (TT) + 2 X (AT) + 2 X (AA)*

*= 24+ 2(68)/ 2(24) + 2(24) + 2(68)*

*= 162/232*

*= 0.69*

Hardy Weinberg Equation

*p2 + 2pq + q2 = 1*

(0.31)^2^ + 2(0.69)(0.31)+ (0.69)^2^ = 1

0.0961 + 0.4278 + 0.4761 =1

The sum of the entries is *p*^2^ + 2*pq* + *q*^2^ = 1, as the genotype frequencies must sum to one.

Hardy Weinberg Equation has been reported in article.

**Comment # 2. Some data in the table1 is confusing. For Glucose, obese vs healthy is 94.5 ± 9.1 vs 94.5± 9.2, but p<0.0001, that won’t be possible. The Total cholesterol in control is 105.5 ± 13.6, but the Triglycerides is 104.5± 11.9, it was also impossible, because the Triglycerides is normally about 30% of the Total cholesterol, in this table, it is more than 90%. I would not report the data if I can’t confirm the accuracy.**

**Response:** Thanks you, yes sure this was serious mistake correction has been made in the table While triglyceroids values has been also corrected by reconsidering units conversion and calculation from initial data sets.

**Comment # 3. According to the date of age, two groups was not age-matched, but the difference of age has not reached a significant level (p>0.12) only. Because the age may have a large influence on BMI, they should consider to be covariates in the correlation analysis or ANOVA analysis.**

**Response;** yes sure age influences BMI of an individual therefore we recruited age, sex matched participants for this study as earlier we mentioned this in the result section but now we have also made it clear in material and methods section.

**Comment # 4. If the authors choose the Dominant model in genotype analysis, it also should be used in the whole manuscript.**

**Response;** Thank you for your valued input we have expanded the applied dominant model to the whole manuscript.

**Comment # 5**. **Two-way ANOVA used in table 1? What is the second independent variable besides obesity? Indeed, a two-way ANOVA with obesity and genotype of Asn107lle should be performed with the dependent variables including BMI, NPS level, and other serum indexes in table1 (if they are corrected). There is no need to compare the NPS level between obesity and control in different genotypes unless there has a significant interaction of obesity and genotype on the NPS level.**

**Response;** We are grateful for your direction, that helped to analyses the data more effectively. Secondly we agree with you and have removed the comparison of NPS level between obese and controls in revised submission.

**Comment # 6. The authors reported rs=0.884, p=0.0004 in the result (what is the meaning of rs?), but in the figure, they show r2=0.1797, p<0.0001? And it also seemed there did not have 116 plots on the figure, please confirmed and provided a more cleared one.**

**Response;**.Yes, thank you again actually it is r^2^ values that was wrong presentation now has been fixed.

**Comment # 7**. **Molecular simulation can only provide a hypothesis that the SNP may influence the interaction of NPS and Asn107lle. But no real data supported the interaction was affected, and furthermore, this effect has an influence on obesity. Indeed, the authors should perform a moderation effect analysis using the genotype as a moderating variable or perform a mediation effect analysis using the NPS as a mediating variable, these analyses can check if the interaction of genotypes and NPS has an effect on obesity.**

**Response;** Yes, we agree with your suggestion, Moderation effect analysis by A.Fhayes really is useful tool for such analyses. We have added section in results and Supplementary tableS2.

**Comment # 8**. **Too many figures. Figure 1 does not need to be provided in the main text; Figure 2 and 3 need to be re-figured according to a new two-way ANOVA analysis; Figure5-7 can be put together into one figure.**

**Response.** Yes right we reorganized the figures accordingly.

**Comment # 9. The simulation is just a prediction; I would not write three long paragraphs to report these results, even longer than the results from real data.**

**Response.** Yes we have summarized the simulation data in revised submission.

**Comment # 10. The RESULTS may be better presented with separate paragraphs accompanied by sub-titles.**

**Response.** Yes we have organized our results accordingly. Thank you
